# Investigation of the Novel Lead of Melanocortin 1 Receptor for Pigmentary Disorders

**DOI:** 10.1155/2014/254678

**Published:** 2014-02-17

**Authors:** Hsin-Chieh Tang, Calvin Yu-Chian Chen

**Affiliations:** ^1^Department of Biomedical Informatics, Asia University, Taichung 41354, Taiwan; ^2^Department of Medicine, China Medical University, Taichung 40402, Taiwan; ^3^Department of Biotechnology, Asia University, Taichung 41354, Taiwan; ^4^China Medical University Beigang Hospital, Yunlin 65152, Taiwan; ^5^Computational and Systems Biology, Massachusetts Institute of Technology, Cambridge, MA 02139, USA

## Abstract

Knowing the role of MC1R in skin tanning can provide a brand new idea to resolve pigmentary disorders. **α**MSH has 13 amino acids and is the most essential pigmentary melanocortin responsible for melanin synthesis. One could utilize the compound library to find lead compounds by virtual screening from peptide database and traditional Chinese medicine (TCM) database@Taiwan. Computational simulation provided a convenient technology to survey potential lead. Ligand-based validation set up the reliable model for molecular dynamics simulation. Molecular dynamics simulation approved the binding affinity and stability of the peptides selected by virtual screening. Thus, we concluded that Glu-Glu-Lys-Glu (EEKE), Glu-Gly-Gly-Ser-Val-Glu-Ser (EGGSVES), and Glu-Glu-Asp-Cys-Lys (EEDCK) were potent lead peptides for MC1R to resolve pigmentary disorders.

## 1. Introduction

Excessive melanin contributes to skin tanning or darkening. Ultraviolet (UV) radiation leads to skin pigmentation by manufacturing melanin in the melanocytes located at the basal layer of epidermis. Expression of the pro-opiomelanocortin (POMC) gene producing *α*-melanocyte stimulating hormone (*α*MSH) takes place in keratinocytes. *α*MSH recognizing melanocortin 1 receptor (MC1R) located on the cell membrane of melanocytes starts a series of signal pathways [1]. The *α*MSH/MC1R triggers downstream signal transduction which is followed by cyclic adenosine monophosphate (cAMP), protein kinase A (PKA), and cAMP responsive element binding protein/cAMP responsive element (CREB/CRE) pathway [2]. Microphthalmia-associated transcription factor (MITF) begins its function in turn. It is an important protein that controls the activation of following melanotic genes: tyrosinase and tyrosinase related protein 1 and 2 (Trp1 and Trp2) [3, 4].

MSHs belong to the POMC hormone groups and include three types of *α*, *β*, and *γ*-MSH [5]. Generally speaking, *α*MSH is a pituitary peptide hormone derived from adrenocorticotropic hormone (ACTH) [6]. *α*MSH which affects skin pigmentation mainly produces locally instead of pituitary origin [7].*α*MSH has 13 amino acids and is the most essential pigmentary melanocortin responsible for melanin synthesis or melanogenesis [8]. Its amino acid sequences are Ser-Tyr-Ser-Met-Glu-His-Phe-Arg-Trp-Gly-Lys-Pro-Val [9]. Synthetic analogs of *α*MSH have been developed as useful probes binding to the melanocortin receptor or MC1R which is overexpressed in melanoma lesions [10, 11]. His6-Phe7-Arg8-Trp9 (HFRW) is the most common active motif approved in the literature [12, 13]. Experimental order exchange of *α*MSH had been demonstrated as high-affinity peptides binding to the MC1R but loss of their agonistic function [14].

Melanocortin receptors belong to class A or rhodopsin of the superfamily of 7-transmembrane G protein-coupled receptors (GPCRs) [15–17]. GPCR receives external signal, for example, hormones and neurotransmitters, vary in molecular size from small peptides to large proteins [18]. There are five known melanocortin receptors, MC1~5R [19]. They have similar structure conformation but participate in unique physiologic functions: pigmentation, adrenal function, cardiovascular regulation, obesity or energy homeostasis, and exocrine gland secretion [20, 21]. MC1R is the irreplaceable target involved in regulating our skin or hair color [22]. The coat color of animals or plumage color of birds is also regulated by MC1R gene [23, 24]. MC1R has 318 amino acids; *α*MSH is its agonist, and agouti signal protein is its antagonist. They determine the phenotype of our skin and hair by producing black, brown eumelanin or yellow, red pheomelanin [25].

Protein sequence and structure analysis by computational simulation have become popular technology in recent decades [26, 27]. We use computational systems biology or *in silico* biology to research the protein-molecule or the protein-ligand interaction [28, 29]. Drug discovery integrates systems biology and informatics called computer-aided drug design (CADD) [30, 31]. The advantages of CADD techniques shorten our time to find appropriate drug compound opposite to traditional biochemistry [32, 33]. Quantitative structure activity relationship *in silico* can tell us the properties between small molecule and target protein [34]. Virtual screening and validations through structure-based or ligand-based analysis constitute to CADD procedures [35, 36]. Virtual screening and data analysis utilize docking and molecular dynamics (MD) simulation [37–39]. How long the compound needs to form stable complex structure with target protein can be predicted by MD [40]. Docking and MD accuracy is relying on a series of statistic or score systems [41]. Ligand-based analysis utilizes mathematical model such as Bayesian algorithm [42, 43]. We can choose best candidates from virtual screening and validations as potential effective drugs [44].

Knowing the role of MC1R in skin tanning can provide a brand new idea to prevent UV darkening [45]. Clinical application of *α*MSH analog is significant in managing certain dermatologic diseases [46]. CADD has been rapidly applied in small molecular drug design [47–50]. Virtual screening of compound database becomes the first and convenient way for CADD [51–56]. Screening peptides for compounds as a drug is a method to design antimicrobial peptides and potent peptides for peptide receptors, such as GPCRs [57–59]. MC1R is a peptide receptor, and peptide design for its agonist and antagonist can be achieved [60]. Virtual screening from peptide database and traditional Chinese medicine (TCM) database@Taiwan *in silico* saves our time to filter the functional compounds [61–63]. We attempted to investigate the lead for MC1R to resolve pigmentary disorders.

## 2. Materials and Methods

### 2.1. Compound Database

To investigate lead peptides of MC1R from peptide library, we downloaded all the peptides from PepBank (http://pepbank.mgh.harvard.edu/) to conduct MC1R lead peptide screening [64].

### 2.2. Data Collection

For the purpose of identifying MC1R lead peptides, we obtained the structures and corresponding bioactivities (pIC_50_) of 18 peptides to construct the data set for ligand-based prediction [65].

### 2.3. Homology Modeling

The MC1R protein sequence was acquired from the Uniprot Knowledgebase (Q01726, MC1R_Human). The 3D structure of human MC4R was acquired from Protein Data Bank (PDB ID: 2IQP). MC1R sequence and the template structure were aligned by Discovery Studio (DS) 2.5. The rational MC1R model was further examined by Ramachandran plot [66].

### 2.4. Structure-Based Virtual Screening

The ligands from PepBank and the control ligand, His-Phe-Arg-Trp (HFRW), were prepared for specified modeling methods. We used Chemistry at HARvard Molecular Mechanics (CHARMm) force field to set up the model [67]. Docking and scoring functions were estimated by LigandFit module in DS 2.5. We applied the scoring functions including Dock Score, piecewise linear potentials (-PLP), and potential of mean force (-PMF) [47, 50].

### 2.5. Ligand-Based Validation

Bayesian network constructed the property of descriptors by integrating the data of training set and test set. The data of descriptors and pIC_50_ were discretized to reduce bias distribution [68]. They were discretized into a maximum of three categories. The training set was defined as linear regression analysis for every pIC_50_ category after data discretization [69]. We used Banjo package and Bayes Net Toolbox (BNT) package for simulation in our study. The 18 ligands were randomly divided into 13 training sets and 5 test sets for further validation.

### 2.6. Molecular Dynamics (MD) Simulation

We used Simulation module in DS 2.5 for MD simulation. The cytoplasmic status was simulated with transferable intermolecular potential 3P (TIP3P) water at 0.9% NaCl concentration. Selected protein-ligand complexes from docking were conducted under minimization, heating, equilibration, and production. The minimization phase included 500 steps of deepest descent and 500 steps of conjugated gradient. The heating time from 50 K to 310 K was 50 ps. The equilibration time at 310 K was 150 ps. The production time with constant temperature dynamics method was 10 ns. The temperature decay time was 0.4 ps. The Analyze Trajectory module was used to analyze total energy, root mean square deviations (RMSD), gyrate, mean square deviation (MSD), and solvent accessible surface (SAS) for each ligand and protein-ligand complex. We also illustrated cluster analysis to observe structure features during MD. Illustration of disordered protein was shown to exclude disordered residues [70, 71]. We used LigandPath module to estimate the possible pathway for each ligand. A surface probe was set at 6 Å, and minimum clearance was set at 3 Å. 

## 3. Results 

### 3.1. Homology Modeling

**Figure 1:**
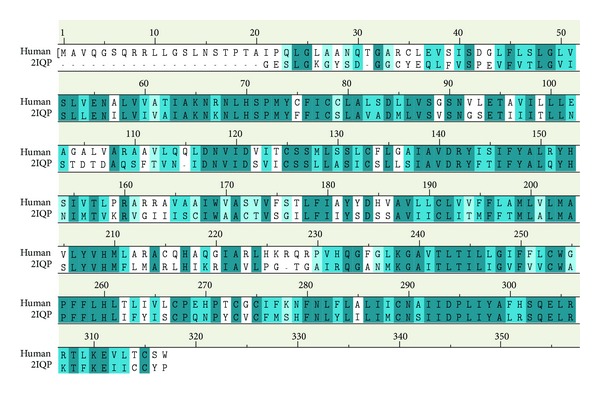
Sequence alignment between MC1R_human and template (2IQP). The identity is 49.8% and the similarity is 72.4%.

**Figure 2:**
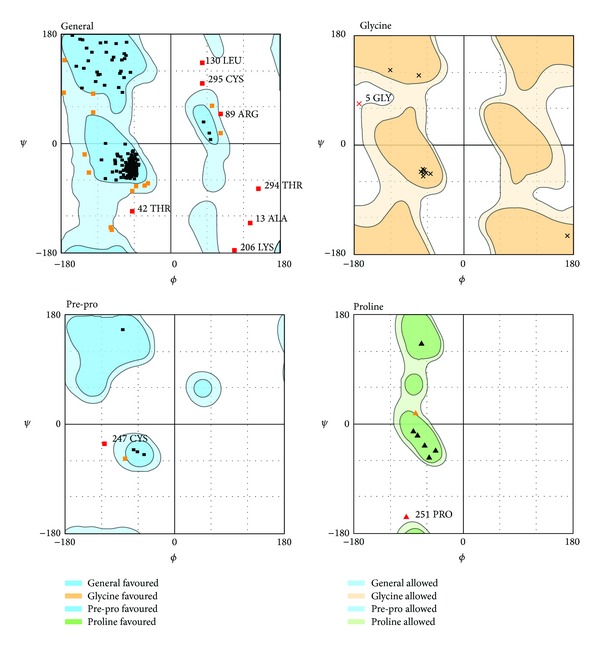
Ramachandran plot of MC1R-modeled structure. Number of residues in favored region (~98.0% expected): 444 (84.7%). Number of residues in allowed region (~2.0% expected): 52 (9.9%). Number of residues in disallowed region: 28 (5.3%).

The overall identity of sequence alignment between MC1R and template was 49.8%. The overall similarity was 72.4% (Figure 1). Ramachandran plot of MC1R-modeled structure indicated that 84.7% of residues were in the favored region, 9.9% were in the allowed region, and only 5.3% were in the disallowed region (Figure 2).

### 3.2. Structure-Based Virtual Screening

**Figure 3:**
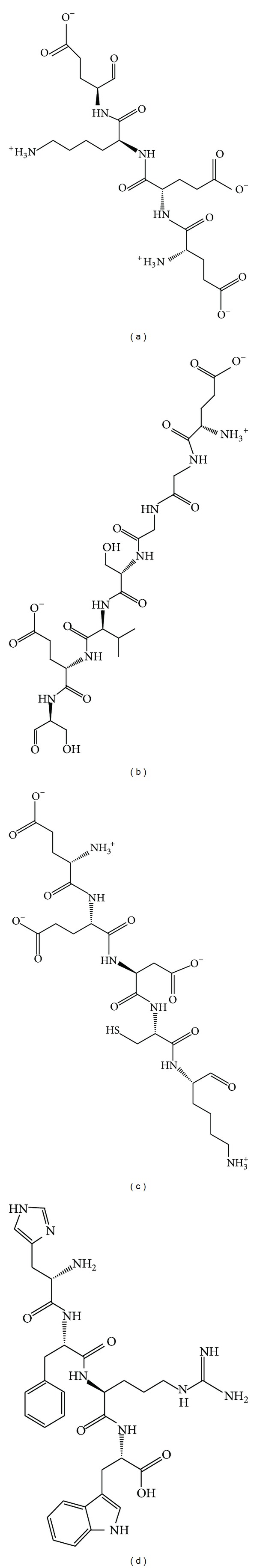
Scaffold of top 3 candidate peptides: (a) EEKE, (b) EGGSVES, (c) EEDCK, and the control: (d) HFRW.

**Figure 4:**
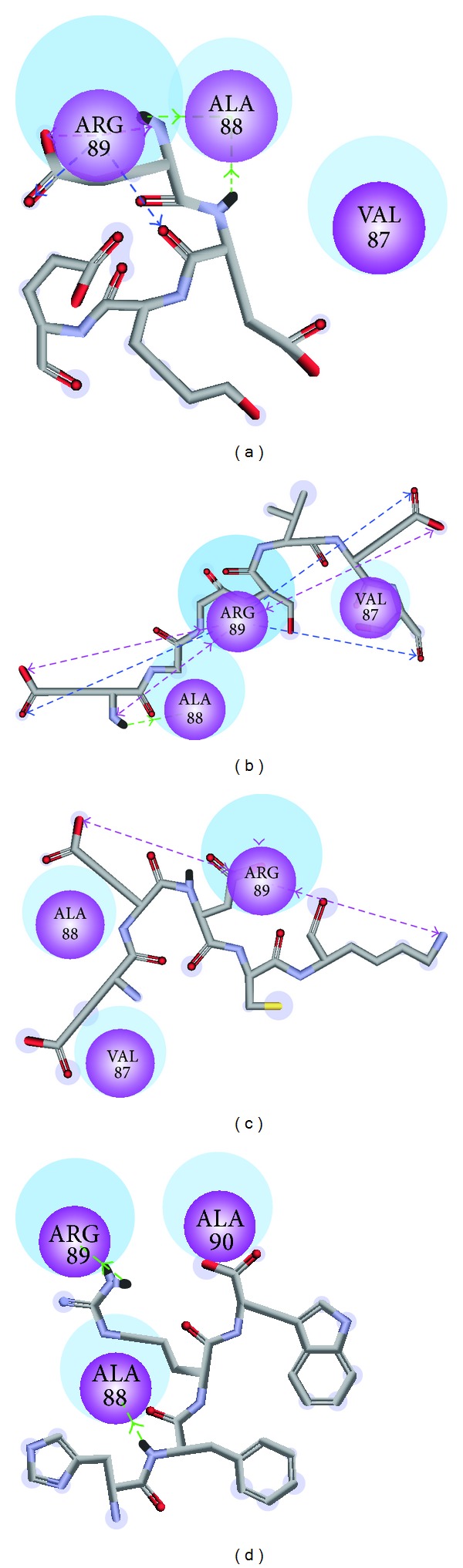
Docking poses of (a) EEKE, (b) EGGSVES, (c) EEDCK, and (d) HFRW. Purple sphere: Residues involved in hydrogen bond (H-bond) or charge interaction. Blue halo around the residue: the solvent accessible surface (SAS) of an interacting residue. The blue halo was proportional to SAS. Green dashed line: H-bond with an amino acid main chain. Blue dashed line: H-bond with an amino acid side chain. Pink dashed line: charge interaction.

Dock score, BNT, -PLP1, -PLP2, and -PMF for the top 10 peptides ranked by Dock score were listed in Table 1. Integrating these data, we selected 3 following peptides: Glu-Glu-Lys-Glu (EEKE), and Glu-Gly-Gly-Ser-Val-Glu-Ser (EGGSVES), Glu-Glu-Asp-Cys-Lys (EEDCK) as candidates for further investigation (Figure 3). Docking poses of EEKE, EGGSVES, EEDCK,and the control (HFRW) with MC1R were illustrated in Figure 4. EEKE formed H-bond with Ala88 and Arg89 and formed charge interaction with Arg89 (Figure 4(a)). EGGSVES formed H-bond with Ala88 and Arg89 and formed charge interaction with Arg89 (Figure 4(b)). EEDCK formed charge interaction with Arg89 (Figure 4(c)). The control formed H-bond with Ala88 and Arg89 (Figure 4(d)).

### 3.3. Ligand-Based Validation

**Figure 5:**
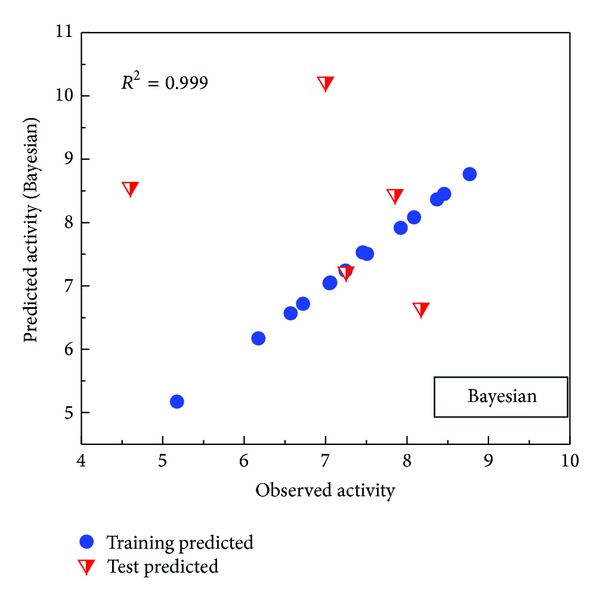
Ligand-based validation: Bayesian network. 18 ligands were randomly divided into 13 training sets and 5 test sets. *R* ^2^ = 0.999.

We illustrated the correlation of observed and predicted activities using the BNT model. The *R* ^2^ value of 0.999 indicated that it is a highly reliable model (Figure 5).

### 3.4. Molecular Dynamics (MD) Simulation

**Figure 6:**
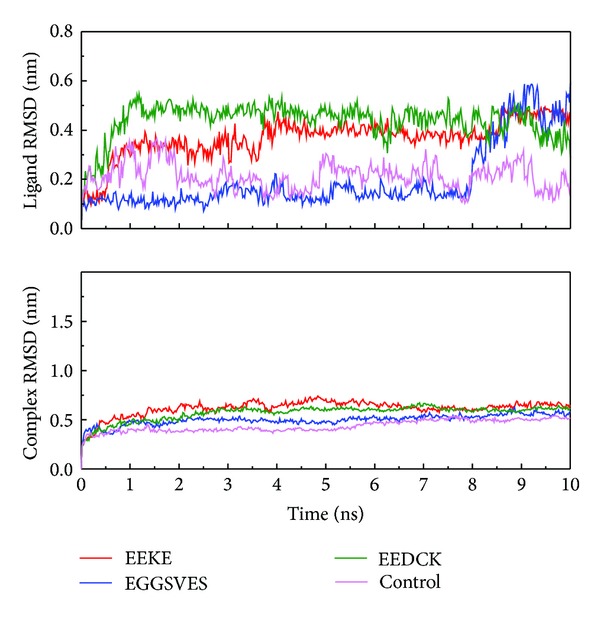
Ligand and complex root mean square deviation (RMSD). Although each ligand alone had larger deviation, the corresponding ligand-protein complex was relatively stable.

**Figure 7:**
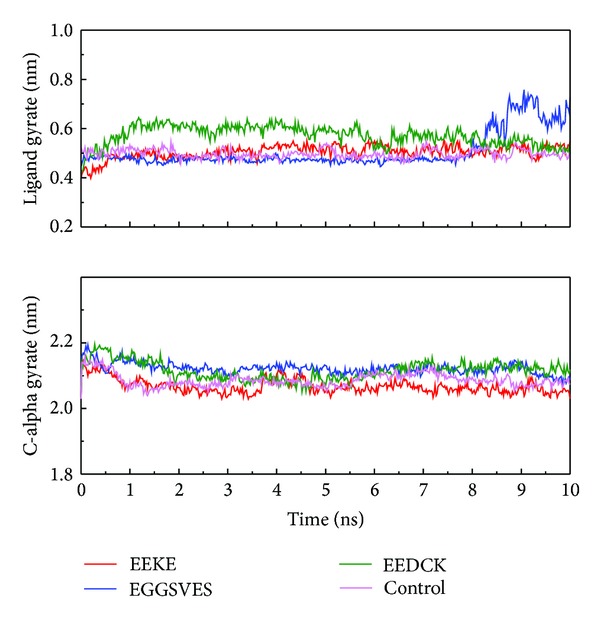
Ligand and C-alpha gyrate. Only EGGSVES had larger gyrate value after 8 ns, other peptides or the complexes were stable in contrast.

**Figure 8:**
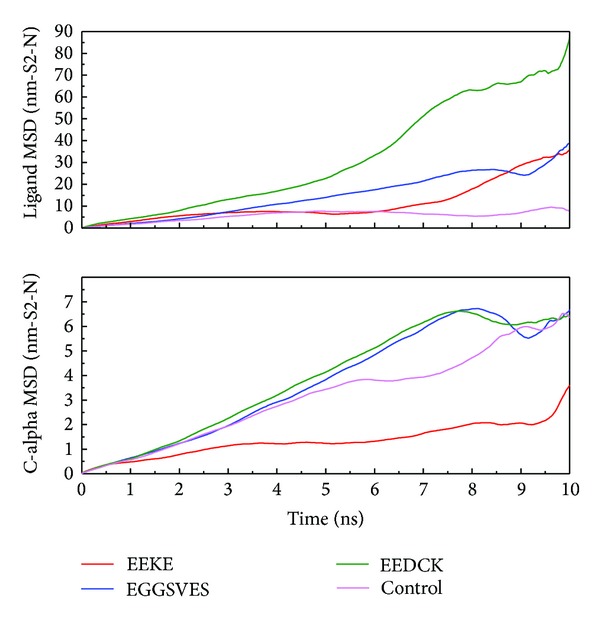
Ligand and C-alpha mean square deviation (MSD). All the ligands and complexes had predictable trends but had larger fluctuation after 8 ns relatively.

**Figure 9:**
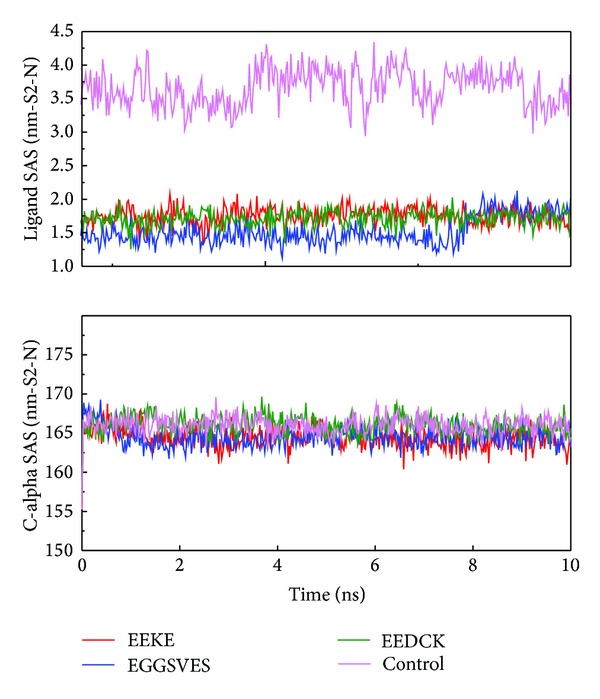
Ligand and C-alpha solvent accessible surface (SAS). The control ligand had larger SAS value compared with other ligands, but its corresponding complex was almost consistent to other complexes.

**Figure 10:**
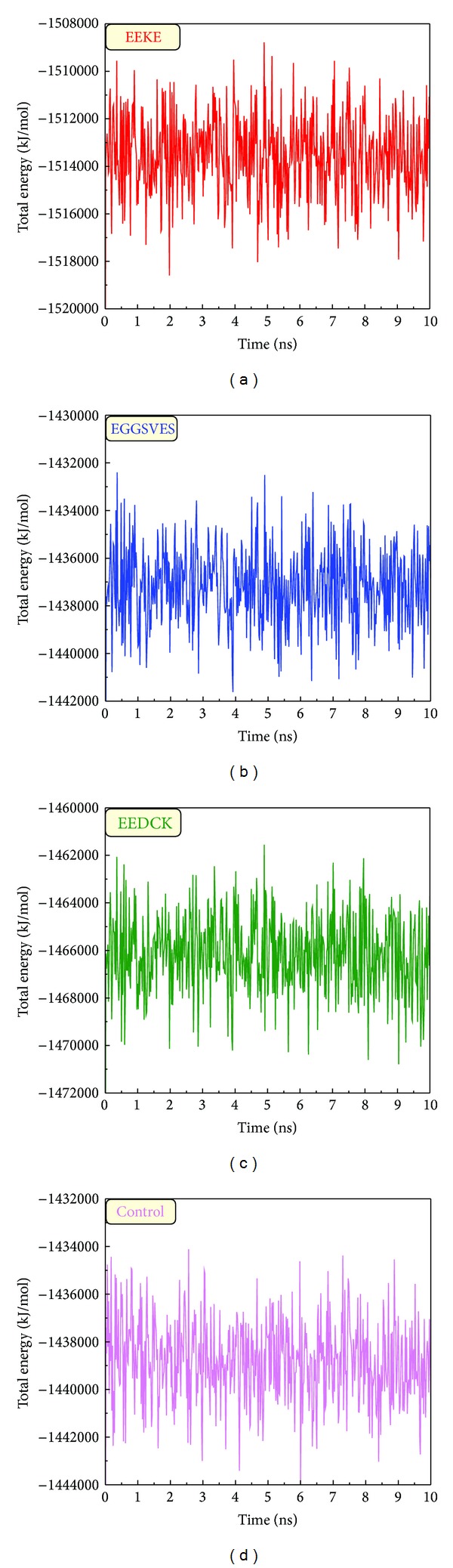
Total energy of (a) EEKE, (b) EGGSVES, (c) EEDCK, and (d) the control (HFRW).

**Figure 11:**
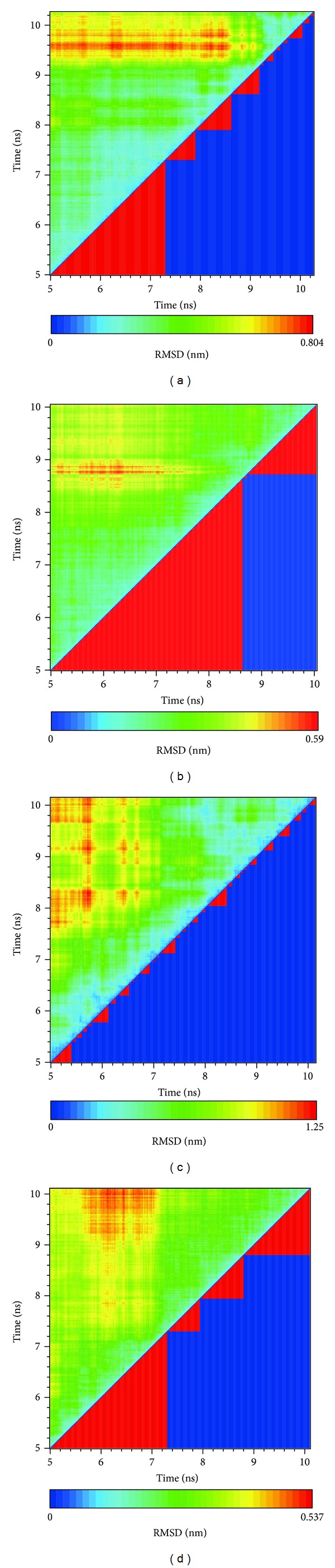
RMSD values (left upper portion) and graphical depiction of clusters (right lower portion). (a) EEKE, (b) EGGSVES, (c) EEDCK, and (d) the control (HFRW).

**Figure 12:**
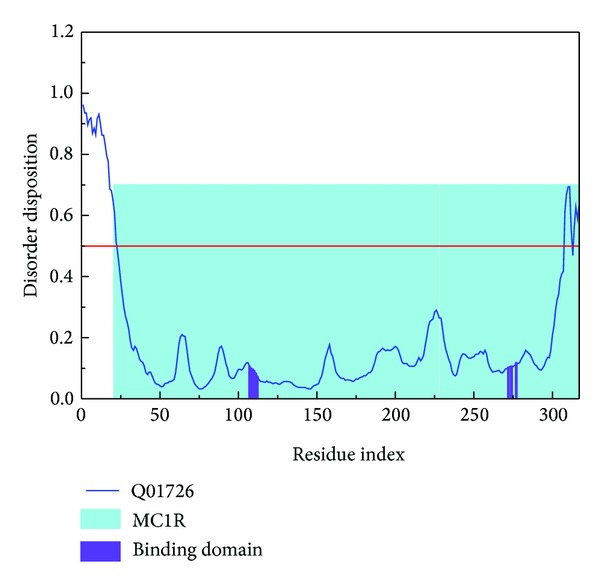
Disorder disposition of MC1R-modeled structure. Most residues are in the non-disordered region (below the red line).

**Figure 13:**
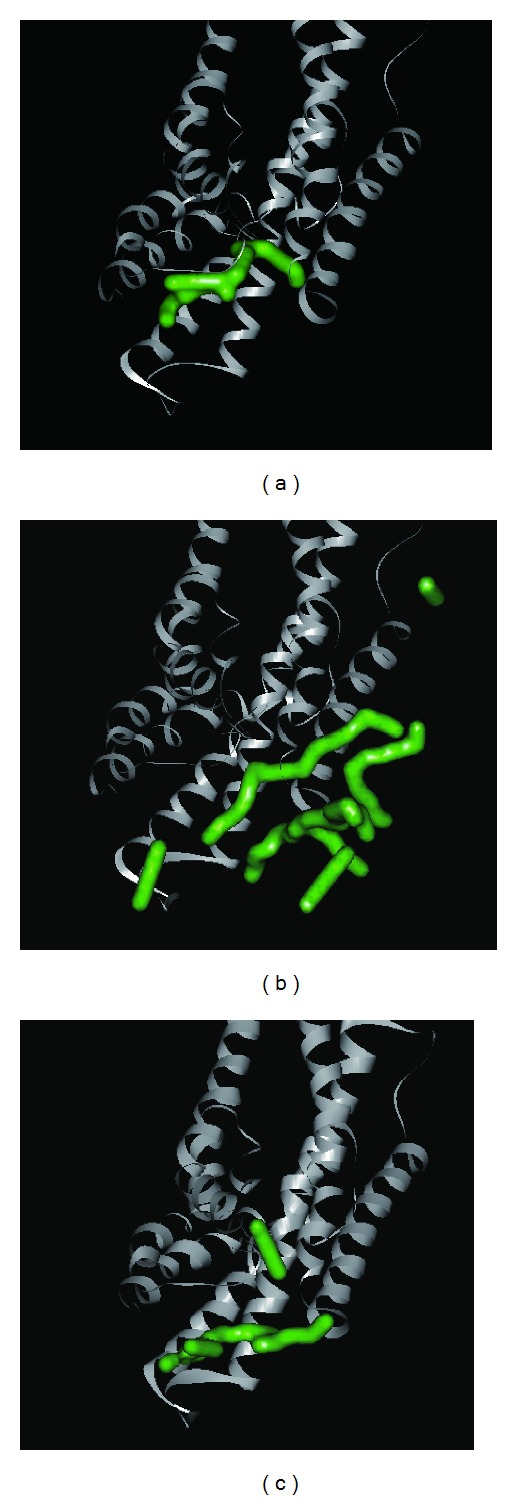
3D simulation of ligand pathway for (a) EEKE, (b) EGGSVES, and (c) the control (HFRW) bound with MC1R. EEDCK is not shown here.

We analyzed MD trajectories which were generated by Gromacs. Root mean square deviation (RMSD) showed the deviation from the starting structure of each ligand or complex to the end of MD. EGGSVES had larger deviation after 8 ns, but the corresponding ligand-protein complex was relatively stable in the same period. Other peptides were stable during 10 ns MD in contrast (Figure 6). gyrate, or radius of gyration, measured the distance of the atoms relative to the center of each mass. gyrate indicated the compact degree of each ligand or complex. Interestingly, EGGSVES had larger gyrate value after 8 ns, but the corresponding complex was also relatively stable. Other peptides were stable during 10 ns MD in contrast (Figure 7). Mean square deviation (MSD) measured the movement of atoms from their initial positions to the end of MD. MSD indicated the trend of each ligand or complex during MD. All the ligands and complexes had different line graphs, but the long-term trends could be predictable (Figure 8). Solvent accessible surface (SAS) measured the surface area of each ligand or complex in contact with the water. Although the control ligand had larger SAS value compared with other ligands, but the corresponding complex was almost consistent to other complexes (Figure 9). In addition, total energy of each ligand or complex means the total energy of atoms during MD. The total energy would fluctuate, but overall trend was consistent (Figure 10). We performed cluster analysis with RMSD values to identify the representative structure of the complex. The cluster analysis could identify two adjacent structure features for each complex during 5–10 ns MD. EEDCK ligand-protein complex had fluctuated structure features, indicated the complex had undergone many tiny changes (Figure 11). Most residues of MC1R-modeled structure were not in the disordered region (Figure 12). Different ligand pathways for EEKE, EGGSVES, and the control bound with MC1R were illustrated. EEDCK was not shown due to out of the criteria mentioned in materials and methods (Figure 13).

## 4. Discussion

### 4.1. Compound Database

MC1R is a peptide receptor, so we utilized the PepBank which was established by Massachusetts General Hospital, Harvard University, to conduct MC1R lead peptide screening. The PepBank database has contained nearly 20000 bioactive peptides. It is a useful and convenient peptide database.

### 4.2. Homology Modeling

The high percentage of identity and similarity of sequence alignment between MC1R (Q01726) and template (2IQP) indicated that the sequence alignment was reasonable. The high percentage of residues in the favored and allowed region indicated that the MC1R-modeled structure was reliable.

### 4.3. Structure-Based Virtual Screening

His-Phe-Arg-Trp (HFRW) is the most common key motif for MC1R. We originally expected the outcome peptide sequence from virtual screening should be similar to the control (HFRW). However, Dock score, BNT, -PLP1, -PLP2, and-PMF of the top 10 candidates were almost better than the control. The top 3 candidates which we selected were EEKE, EGGSVES, and EEDCK. Their sequences were quite different from the control. We could speculate that these candidates at least had similar function as the control and might have better affinity for MC1R. Comparing the docking poses of the top 3 candidates and the control with MC1R had the common finding. All of EEKE, EGGSVES, and the control formed H-bond with Ala88 and Arg89. All of EEKE, EGGSVES, and EEDCK formed charge interaction with Arg89. We could conclude that Ala88 and Arg89 were key residues for the top 3 candidates and the control.

### 4.4. Molecular Dynamics (MD) Simulation

RMSD, gyrate, MSD and, SAS were utilized to analyze the stability of each ligand or complex. Although some ligands were not stable during MD, the ligand-protein complexes were stable relatively. So either of EEKE, EGGSVES, EEDCK, or HFRW could form stable complex with MC1R.

Further analyzing the figure of ligand RMSD, EEDCK had the largest average deviation than other candidates. Interestingly, the deviation of EEKE and EGGSVES exceeded EEDCK after 9 ns. However, analyzing the figure of complex RMSD, EEKE had the largest average deviation than other candidates. Although EGGSVES alone had larger deviation at 8-9 ns, the EGGSVES-MC1R complex did not have substantial change at the same time. It showed that deviation of individual ligand did not affect the stability of its corresponding complex.

Further analyzing the figure of ligand gyrate, EEDCK still had the largest average value than other candidates. Although EGGSVES alone had larger fluctuation at 8-9 ns, the EGGSVES-MC1R complex (c-alpha) did not have substantial change at the same time. It showed that gyrate of individual ligand did not affect the stability of its corresponding complex, either.

From the additional analysis of the figure of ligand MSD, EEDCK still had the largest deviation than other candidates. However, from results of the complex (c-alpha) MSD, EEKE had the lowest average deviation than other candidates. We speculated that EEKE-MC1R complex vibrated back and forth contributing to distinct presentation in complex RMSD and c-alpha MSD. EGGSVES vibrated in opposite direction might explain the upward slope in ligand RMSD and the downward slope in ligand MSD at 8-9 ns.

Further analyzing ligand SAS, the control had the largest average value than the 3 candidates. The result might be related to the hydrophobic side chain of the control.

Further analyzing the figure of total energy, EEKE had the lowest total energy (−1514000 kJ/mol), and EGGSVES had the highest total energy (−1437000 kJ/mol). We speculated that EEKE-MC1R complex only vibrated back and forth contributing to the lowest total energy.

When individual ligand bound to MC1R, MD was convenient to analyze the change of the ligand or the ligand-protein complex. MD could evaluate whether the ligand or the complex was stable or not under dynamic condition. MD might help us understand what happened during conjugation of the ligand and protein. Comparing the figures of RMSD and gyrate, EGGSVES had larger change after 8 ns. The change implied that the structure of EGGSVES underwent some kind of deviation or even translocation. This change did not affect the conjugation of EGGSVES with MC1R because the ligand-protein complex was stable relatively. Comprehensive assessment of the methods of MD, such as RMSD, gyrate, MSD, SAS, and total energy, indicated that all the 3 candidates and the control could form stable complexes with MC1R [72–81].

## 5. Conclusion

MC1R is important for skin tanning. *α*MSH is a melanotropin which can bind to MC1R. His-Phe-Arg-Trp (HFRW) is a key motif for conjugating with MC1R. We tried to find potential peptides that can also bind to MC1R by virtual screening of peptide database. Glu-Glu-Lys-Glu (EEKE), Glu-Gly-Gly-Ser-Val-Glu-Ser (EGGSVES), and Glu-Glu-Asp-Cys-Lys (EEDCK) had better affinity for MC1R. The binding affinity was further validated by molecular dynamics. Thus, we concluded that EEKE, EGGSVES, and EEDCK were more potent lead peptides for MC1R to resolve pigmentary disorders.

## Figures and Tables

**Table 1:** Top 10 candidates of scoring function from PepBank database screening.

Name	Dock score	BNT	-PLP1	-PLP2	-PMF
EEKE	345.464	7.963191	37.91	36.24	32.59
EGGSVES	338.21	6.979679	32.61	40.4	41.39
EEDCK	337.005	9.512784	24.46	26.69	26.37
GEGEGSGG	335.016	9.346013	42.84	51.79	29.34
SEEEAA	322.144	12.351714	58.12	67.98	24.42
DSGVETS	316.584	8.611215	13.43	19.48	31.47
EGEVGLG	315.171	5.676182	28.59	30.23	22.78
EAGVDAA	315.157	7.491065	24.74	25.56	30.21
DTAGQE	311.701	6.382156	33.58	29.06	34.39
EEKE	311.503	8.708929	44.54	48.58	27.17
His-Phe-Arg-Trp (HFRW)*	97.949	3.431413	32.8	26.9	28.15

*Control.
